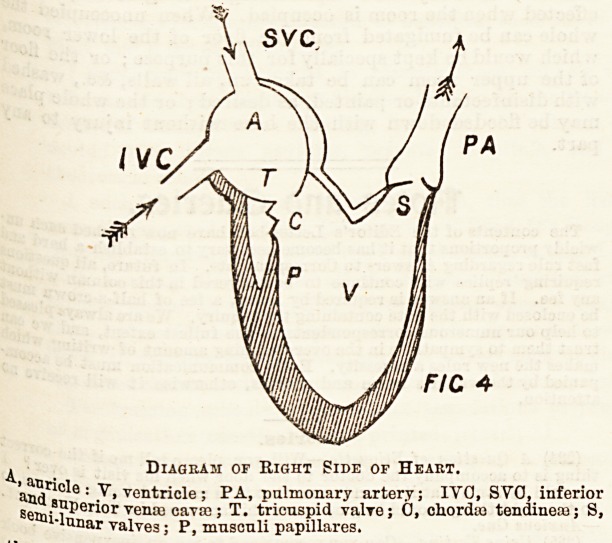# The Hospital Nursing Supplement

**Published:** 1895-08-31

**Authors:** 


"The Hospital" Nursing Mirror.

Being the Extra Nursing Supplement of "The Hospital" Newspaper.

[Contributions for this Supplement should be addressed to the Editor, The Hospital, 428, Strand, London, W.C., and should have the word "Nursing" plainly written in left-hand top corner of the envelope.]

### News from the Nursing World.

### OUR SICK AND AGED POOR.

Our sick poor may be congratulated on the tone adopted by leaders on both sides of the House of Commons in regard to Poor Law administration. Speaking of the classification of paupers and of workhouses, Mr. Chaplin thought that much might be done for the paupers in that direction, and in this every one who has knowledge of what goes on in workhouses will agree with him. Bad as it may be to bear with official bumbledom, the purgatory through which the respectable aged poor pass on their way to the peace of death is not so much due to callous officialism as to bad classification and to enforced association with those who are proof against all rules of decency in conduct or in speech. There can be no doubt that classification is the first essential for ensuring comfort in old age for those poor but decent folk who have worked as hard as any, and whose sole crime it is that—often through the fault of others—they have failed. Mr. Chaplin also said that he would cordially continue the policy of the department, which discouraged, so far as possible, the employment of inmates as nurses, and sought to obtain an efficient performance of the duties by means of institutions like the Workhouse Nursing Association. As an official statement this is most satisfactory. Sir Walter Foster spoke on the question of nursing, showing how the Local Government Board had stimulated the local authorities to attention to this matter, and he pointed to the possibility of some future action in the direction of grouping of unions, and consequent classification of workhouses and infirmaries, so that there might be a house for the more deserving poor, another for the sick poor, and possibly another for well-behaved women and children. All this, again, is well, but let us plead for the infirm. Signs of the times point to the probability that the really sick even among the very poor will sooner or later obtain that proper care and nursing which they require; but the more the sick are relegated to a separate class the more the infirm tend to be cut off from that influence for good which arises from the presence of a trained nurse even under the same roof. We hope that, whatever is done for the sick, the aged and the infirm will not be forgotten. When strength has gone and illness has not yet come to qualify for the infirmary, the lot of the aged and infirm entrusted to pauper helps is of all fates the least desirable.

### NURSING SCHOLARSHIPS.

There seems a danger of the nursing scholarships granted by some County Councils forming a fresh channel whereby the ranks of partially trained nurses will be recruited. Probably this is far from being the intention of the promoters of the scheme, but, according to report, it is nevertheless one of the results. Candidates selected for training are granted a certain instruction in maternity work, board, lodging, fees, fares, and other personal expenses being also defrayed for them. Their services are not invariably claimed in their native villages at the end of this time, but these women can, it appears, without let or hindrance, start as unsupervised nurses on their own account wherever they like. Therefore, it seems as if the County Councils, so far from considering the special interests of the poor in a particular district, invest money, which might ensure the thorough training of a few picked candidates to provide terms of instruction for women who, on the strength of it, pose and practice as "nurses."

### ANOTHER UNTRAINED NURSE.

The Truro Guardians, by a majority of forty-eight to six, have deliberately appointed an untrained woman as nurse to the sick poor in their workhouse. The *Cornwall Gazette* protests most emphatically against such a decision following the promise made by the Guardians to the Local Government Board that a trained nurse and other improvements should be introduced into the workhouse. This appointment is hardly likely to obtain the sanction of the Local Government Board; but it is to be regretted that it should have been completed by those responsible for the care of sick paupers.

### MRS. BROWN'S POULTICE.

"Good morning, Mrs. Brown," said the district nurse; "I've come to put another poultice on Jimmie." "Thankee kindly, nurse," responded Mrs. Brown, "but us shan't need to trouble you to make any more for him!" "But I've just seen the doctor, and he wishes the poultices continued for another day or two," was nurse's reply. Mrs. Brown smiled. "Yes, that's all right, but I've learnt to make 'em myself!" she said with a good deal of pride. "That's a good thing," remarked nurse rather doubtfully, "but I understood you were quite set against treating the wound yourself; you know you told me so only yesterday when I offered to show you how to do it." Mrs. Brown nodded her head affirmatively, and then explained, "Well, you see, nurse, it was this way, I heard the lady from London lecturing last night; I wasn't thinkin' of goin', but the vicar's wife asked me, and I didn't like to disappoint her, and so I went. Well the lady told us just everythin' about nursing! there wasn't nothin' as she didn't talk about, and say as it was quite easy to do. So, thinks I, I'll just give nurse a surprise in the morning!" "May I just have a look at Jimmie's leg?" asked nurse gently. Proudly his mother exhibited to the trained district nurse cotton wool, macintosh, and linseed duly proportioned, but, unfortunately for the victim of her first essay, Mrs. Brown had laid the wool on the wound and covered it with the macintosh. Over this, quite exposed to the air, was placed a heavy, tepid, hard mass of linseed!

### THE VALUE OF TESTIMONIALS.

There being very few callings open to women in the present day wherein a good place can be taken without some sort of testimonials or certificates, the demand for which creates a supply of more or less valuable manuscript; amongst the latter, conspicuous from its frequency, may be noted the undated testimonial, the obvious absurdity of which needs no comment. Even duly dated and signed testimonials are sometimes accompanied by odd communications. An example reached us the other day in the case of a nurse who, doubtless wishing to give a favourable impression of her talents, wrote that her hospital certificate of training "qualified her to lecture and teach the theory of nursing." At all times printed or copied testimonials gather value when accompanied by a recommendation from some reliable person to whom the applicant is known, rather than by comments of a self-laudatory nature.

### TAUGHT TO WORK.

Instruction in cutting out, dressmaking, house-work, cooking, baking, washing, dairy work, poultry and bee keeping, is given to the pupils at the Leaton Colonial Training Home in Shropshire. Ladies desiring to go out as companion-helps to the colonies are received at moderate fees, and also young girls to be trained as colonial servants. The home is certified, and can therefore receive children from the Local Government Board, as well as orphaned or deserted girls who have not been in the workhouse. The home, established in 1890, is superintended by honorary workers, and is supported by pupils' fees, supplemented by subscriptions and donations from persons interested in the practical education of women and girl emigrants.

### REGISTRATION AND PENSIONS.

Registration and pensions for nurses having become matters of public interest for the last seven or eight years, it is refreshing to find anyone to whom these subjects are new. A gentleman writing from the Isle of Wight to a medical contemporary seems to think nothing has yet been done in either direction, for he remarks: "I believe that a simple system of registration of nurses by the medical profession is the only method of obtaining control," and appears to imagine this can be easily managed by the branches of the British Medical Association. He owns that serious additional labour might be involved in judging credentials of candidates for registration, and possibly a study of the methods of training schools for nurses would still further impress this on his mind. The difficulties which individual institutions find in keeping a register of their own nurses alone, and the unremitting care needed to keep these registers up to date, are very great. The Royal British Nurses' Association has struggled bravely for years, officered by a large professional executive, to supplement these existing registers; but their own members are obliged to own that, after all their trouble, names stand on their register which ought not to be there. If the voluntary labour of years in this direction has resulted in comparative failure, the "district branches" of the British Medical Association can hardly be expected to sift credentials with any prospect of immediate success. As regards the question of control, it was settled in the earliest days of training, and the primary lesson taught to every probationer is that of implicit obedience to a doctor's orders. Neither is State registration likely to find general favour. After all, nursing is merely the handmaid of medicine; and, in a good nurse, qualities are demanded which can be no more registered than those of good home daughters, wives, or mothers. Neither in the matter of pensions is the gentleman in the Isle of Wight in touch with the times, otherwise he would hardly consider it incumbent on him to suggest that the medical profession should raise £800 a year for the future provision of nurses found worthy of a place on this ideal register, while a Royal National Pension Fund for Nurses already exists, and possesses an invested capital of £200,000.

### AN AMERICAN TRAINING SCHOOL.

The training school connected with the University Hospital at Ann Arbor, Michigan, was instituted in 1892. Seven nurses have already completed their training and received diplomas, whilst twelve are now in course of training. Three male attendants are also undergoing instruction in the hospital, and like the female pupils appear to have facilities for securing experience in every branch of trained nursing.

### RECEIPTS AND EXPENDITURE.

In comparing the fees of nurses in different countries, an English woman is apt to think herself worse paid at home than some of her sisters abroad. In America, for instance, private nurses get twice or three times as much money as in England, but clothes there are far more expensive, and so, as a rule, is the cost of living. A nurse generally works single-handed, and with a case of serious illness is practically never off duty, getting such sleep as she can in the patient's room Domestic servants being in the States inadequate in quality and quantity, both cooking and housework must fall on the nurse, who generally prepares all her patient's food until convalescence enables him to again share the family meals. Fees are, therefore, earned at an expenditure of strength and health which cannot long be continued by the average woman. On the Continent, English nurses are not, as a rule, recipients of high pay, in spite of which temporary engagements for the winter season are eagerly sought in the Riviera. The salaries paid in the Colonies sound good, but the work is often very hard, and if health or circumstances compel a nurse to break her contract with an institution which paid her fare out, she has herself to bear the heavy cost of her return journey. Other expenses to be taken into account are those of outfit, the allowance for uniform not covering the numerous articles of wearing apparel which a nurse needs when going away for several years. On the whole the earnings of a first-class nurse in England compare favourably with other countries.

### SHORT ITEMS.

A very successful fête was held in the Hasfield Court grounds on the 15th inst., in aid of the District Nursing Fund. Various amusements were provided, a "sale of work" being among the attractions, and the sum of £52 17s. was made.

### Elementary Physiology for Nurses.

By C. F. Marshall, M.D., B.Sc., F.R.C.S.

### IV.—THE CIRCULATORY SYSTEM—(*continued*).

### The Valves of the Heart.

There are (1) the auriculo-ventricular valves, between the auricles and ventricles; (2) the semi-lunar valves, between the ventricles and the great arteries. Let us consider the two sides of the heart separately.

The right auriculo-ventricular, or tricuspid valve. This is situated in the opening between the right auricle and ventricle, and consists of a fibrous ring round the aperture, with three flaps round the margin hanging into the ventricle. The edges of the flaps are attached by strong cords—the chordæ tendineæ — to the walls of the ventricle, or, more correctly, to the tops of muscular projections from the walls—the musculi papillares. The action of the valve is as follows. The auricle contracts first and fills the ventricle, the valve offering no resistance in that direction. The ventricle then contracts, and the blood, getting behind the valve, drives it up so as to close the aperture between the ventricle and auricle, the valves being prevented by the chordæ tendineæ from being driven into the auricle. The ventricle during contraction becomes, as a whole, shorter, so

Diagram of Right Side of Heart.

A, auricle: V, ventricle; PA, pulmonary artery; IVC, SVC, inferior and superior venæ cavæ; T. tricuspid valve; C, chordæ tendineæ; S, semi-lunar valves; P, musculi papillares.

that the valves would have a tendency to be driven into the auricle during the last part of the contraction, if they were not counteracted by the contraction of the musculi papillares, which have the effect of shortening the chordæ tendineæ.

The semi-lunar valves are at the base of the pulmonary artery, and allow the blood to pass readily from the ventricle into the artery, but stop it flowing the other way.

On the left side of the heart we have similarly the mitral valve between the left auricle and ventricle, and the semi-lunar valves at the orifice of the aorta, or main artery of the body. They act in precisely the same manner as the valves on the right side of the heart which we have just considered. The action of these valves is readily demonstrated on the heart of a sheep by pouring water through the auricle into the ventricle and noting the floating up of the auriculo-ventricular valve. If we squeeze the ventricle the valves will act perfectly. So with the semi-lunar valves, if we pour water into the aorta or pulmonary artery they will close at once.

The contractions of the heart occur regularly, rather oftener than once a second, and are rythmical. The auricles contract first and simultaneously; the ventricles follow afterwards, also simultaneously. Then occurs a pause, during which the auricles are refilling.

The sounds of the heart may be heard either directly by applying the ear to the chest, or indirectly through the stethoscope. The first sound is long, dull, and booming; the second is short and sharp. The two sounds are usually said to be represented by pronouncing the two syllables lubb-dup. The first sound occupies about half the cardiac cycle, and corresponds to the contraction of the ventricles; it is caused partly by muscular contraction, and partly by the vibration of the auriculo-ventricular valves. The second sound occupies one-fifth of the cardiac cycle, and is due to the closing of the semi-lunar valves.

The cause of the rhythmical contractions of the heart is in the heart itself, for if we cut out the heart of a frog it may be kept beating for a day or longer. Further, if we cut up a frog's heart each auricle and each half of the ventricle (for the frog's heart has only one ventricle) will contract independently. This contraction is under the influence of the nervous system, and there are two sets of nerves supplying the heart; the one set, if stimulated, cause it to beat faster, the other set cause it to beat more slowly, or even to stop. Fainting or even death may be caused by sudden joy or grief by stimulating the latter set of nerves.

### The Blood.

If we examine a drop of blood under the microscope, we see (1) a colourless fluid—the liquor sanguinis or plasma; (2) the corpuscles. The corpuscles are of two kinds—(*a*) the red corpuscles, which are circular flattened discs $\frac{1}{3200}$ of an inch in diameter. They are yellow when seen singly, but red in masses. In a cubic inch there are no less than 70,000,000,000. (*b*) The white corpuscles are much fewer in number, and rather larger in size ($\frac{1}{2500}$-inch). They are granular lumps of protoplasm like an amœba. Chemically, the blood is alkaline, and consists of water and solid and gaseous matters. The red corpuscles contain hæmoglobin—a peculiar substance containing iron—which causes the red colour of the blood. The gases in the blood are oxygen and carbonic acid, which we shall consider in the next lecture. The blood in the arteries and veins will necessarily differ, for it is clear that the blood in the arteries will have more oxygen and less carbonic acid than that in the veins. Hence the difference between venous and arterial blood. Also there are differences in the blood from various parts of the body, which we shall consider later.

### Appointments.

[It is requested that successful candidates will send a copy of their applications and testimonials, with date of election, to The Editor, The Lodge, Porchester Square, W.]

Taunton Isolation Hospital.—Miss Rose Reynolds has been appointed Matron of this hospital. She was trained at the Birmingham City Fever Hospital, and acted as charge-nurse there for 2½ years. She goes to Taunton from the Linacre Fever Hospital, Liverpool, where she has been senior sister during the past year. Whilst there she had charge of two blocks with 50 beds, and acted for the matron in her absence. Her testimonials, which are excellent, all agree as to her admirable qualifications as a nurse and as to her powers of administration. We wish Miss Reynolds all success in her new appointment.

### Examination in Hygiene.

Nurse Agnes Mackie, of the Clifton Nurses' Co-operation, is amongst the successful students who passed the examination in hygiene in connection with the Science and Art Department of South Kensington, held in May.

### Everybody's Opinion.

[Correspondence on all subjects is invited, but we cannot in any way be responsible for the opinions expressed by our correspondents. No communications can be entertained if the name and address of the correspondent is not given, or unless one side of the paper only be written on.]

### A SCHEME FOR A SICK ROOM (OR HOSPITAL ROOM).—A SUGGESTION.

A correspondent writes: The necessity for, and the advantages which would accrue from, the possession of a "sick-room" perfect in design and construction will be admitted by every physician, architect, and householder. Although perfection is impossible, granted the requirements of such a structure are known, the scheme which supplies them in the most approved manner will be acknowledged to be the nearest approach to perfection. Isolation, convenience, ventilation, change of atmosphere, temperature, sanitary and disinfecting arrangements, are concisely a few of its requirements; and I trust by explaining this scheme, which is a suggestion for the consideration of the physician, architect, and householder, to demonstrate the possibility of supplying these and other requirements on an improved system. The size, position, and aspect of such a room must be determined by the physician and the architect on the spot, in accordance with the circumstances and requirements of each case. But we will suppose the room and all its necessary appendages to be built as a wing, connected by a passage to a convenient part of the mansion. This passage could easily be constructed and ventilated so as to isolate the room sufficiently; and the length of the passage would be sufficient to set the wing clear away from the wall of the mansion, so that it would have four walls, which is of importance, because of changes of wind, &c. The first floor level would be the level of the floor of the "sick-room"; and below this level from the ground floor level upwards, the building would be constructed as follows, viz.: The inner surface of the walls and the floor would be cemented, or otherwise constructed of materials which could be thoroughly washed without injury, with a hose if necessary. Perfect drainage from the surface of the floor would be provided, without cesspools or any stoppage to the free flow of water. Commencing about three feet below the level of the floor of the "sick room" above, the four walls would, for about two feet of their height, be as perfectly perforated as possible, and be so constructed that the openings could be closed or opened as required, to suit change of wind or other circumstances. Immediately above this two feet of open work would be a system of hot-water pipes arranged horizontally so close together underneath all the floors of the "sick-room" above that air could not ascend between them without being either heated or cooled as required. The cooling in hot weather would be effected by keeping the pipes full of cold water and changing it as required. The door into this lower compartment would be from the outside, and one or two windows would be sufficient, according to the size of the place. Above the hot-water pipes would be light iron girders, arranged to suit the floor of the "sick room" above, which floor would consist of open gratings in suitable sized pieces, as open as possible, but sufficiently close and even to form a good floor. The skirting-board would also be iron. All iron-work up to this point (except the pipes) may either be galvanised or painted, but the carpet, without which the floor would not be complete, would be galvanised iron ⅛ in. to ¼ in. thick, in convenient-sized pieces, and as thoroughly perforated as possible, so as to admit the air freely, but the perforation would be so equal and so fine that perceptible draught would be impossible. If this cannot be accomplished with galvanised iron, wire-cloth may be used; but the fact that the former could be so easily cleansed and disinfected will be its greatest recommendation. Perforated linoleum could be laid above the perforated iron on any part of the floor as the physician desired. The door and windows of the room would be made as perfectly air-proof as possible when shut. The windows would be fitted with close-fitting shutters outside and inside, so that the room could be darkened when a dark room is required; and also so that the windows might be protected from severe weather and high winds when necessary. A perfect system of ventilation would be placed in the roof in accordance with the architecture. The walls would all be cemented and painted with oil-paint, and everything, as far as possible, made of materials which would wash without injury. The boiler would be fixed some distance away from the room, so that noise and dust, &c., would be unknown in the vicinity of the "sick-room." The boiler would be one easily regulated to a nicety, with slow combustion. The heating surface of the pipes being proportionately great, very gentle heat, if regular throughout, would be sufficient. If the above description is sufficiently lucid it will demonstrate the possibility of supplying by this scheme the requirements indicated, viz.: Isolation, by the length and construction of the connecting passage; convenience, by making the passage where it will suit the arrangements of the "mansion"; temperature, which may be high or low as required, without the drying heat which would result from a limited heating surface; sanitary and disinfecting arrangements, easily effected when the room is occupied. When unoccupied the whole can be fumigated from the floor of the lower room, which would be kept specially for this purpose; or the floor of the upper room can be taken up, all walls, &c., washed with disinfectants or painted, as desired; or the whole place may be flooded down with the hose without injury to any part.

### Notes and Queries.

The contents of the Editor's Letter-box have now reached such unwieldy proportions that it has become necessary to establish a hard and fast rule regarding Answers to Correspondents. In future, all questions requiring replies will continue to be answered in this column without any fee. If an answer is required by letter, a fee of half-a-crown must be enclosed with the note containing the enquiry. We are always pleased to help our numerous correspondents to the fullest extent, and we can trust them to sympathise in the overwhelming amount of writing which makes the new rules a necessity. Every communication must be accompanied by the writer's name and address, otherwise it will receive no attention.

### Queries.

(234) *A Question of Etiquette.*—Will you please tell me if the correct thing is to accompany the doctor to the door when his visit is over? I am told by some that it is, and some that it is not, and being anxious to do the right thing should be glad to see this answered in The Hospital. —*Anxious One.*

(235) *Urine Testing.*—Can you recommend to me an inexpensive book with clear, concise instruction on the testing of urine?—*Enquiring Nurse.*

(236) *Massage.*—Will you inform me of a good school for learning massage?—*Nurse.*

(237) *Disinfection by Steam.*—Can you tell me of a work on this?—*H.S.*

(238) *Colonies.*—Is there such a place as the Colonial Institute for Training Nurses to go abroad?—*L. R.*

(239) *Fever Nurses.*—Can anyone tell me of a nurses' hotel where nurses from fever cases can be admitted for a week on moderate terms? —*Private Nurse.*

### Answers.

(234) *A Question of Etiquette* (*Anxious One*).—It certainly is neither customary nor right that a nurse should "accompany the doctor to the door when his visit is over." The nurse should, if possible, come just outside the door of the sick room with the medical man on his departure, so as to give him an opportunity of making any remark that he might not care to make in the patient's presence, but she certainly should not accompany him downstairs; firstly, because she is on duty, and should not leave her patient; and, secondly, because the friends would be sure to resent being thus deprived of the opportunity of themselves speaking to the doctor.

(235) *Urine Testing* (*Enquiring Nurse*).—Any text book on medicine will give the information There is a small book by Dr. Legg, "A Guide to the Examination of the Urine" (Lewis, 3s. 6d.), devoted entirely to the subject, but it contains much more than a nurse is likely to want. We have had several enquiries lately about this matter, and an article will shortly appear in "The Mirror," giving such details as are likely to be required by a nurse.

(236) *Massage* (*Nurse*).—Mrs. Creighton Hale, 89, Mortimer Street, Cavendish Square, is an experienced teacher.

(237) *Disinfection by Steam* (*H. S.*).—We do not know of one, but you might refer to Dr. Parsons' report to the Medical Officer of the Local Government Board in 1884 on disinfection by heat.

(238) *Colonies* (*L. R.*).—No. The training a nurse receives at a good general hospital in England renders her fit to occupy a post abroad.

### Alumnæ Associations.

Read at American Superintendents' Convention by Miss S. F. Palmer, Late Superintendent of Garfield Hospital, Washington, D.C.

In gathering up material for this report on Alumnæ Associations, I have succeeded in obtaining from various sources a list of one hundred and sixty-four training schools—twenty in Canada and one hundred and forty-four in the United States. To the superintendents of these schools I addressed a circular card of inquiry, and received personal answers from one hundred and nine; the remaining fifty-five did not respond.

In a number of instances I had sent communications to hospitals having no training schools, and the superintendents or matrons of these hospitals wrote me very courteous letters informing me of my mistake. I think I am justified in concluding that those superintendents who did not respond have no alumnæ to report, and are not interested in the subject, for in almost every instance of superintendents reporting no organisation, some explanation is offered or regret expressed. A number of schools in this list have not yet graduated a class; in others the number of graduates is small and very much scattered; and in several cases superintendents were waiting to obtain information on the subject before taking active measures for organisation.

The laws of Canada require a special permit from the Government for the organisation of beneficial societies of any kind, and a fee of 100 dollars.

I have made no attempt to classify these schools with reference to their eligibility for membership in the Superintendents' Association, excluding, however, the schools connected with insane asylums, private hospitals, and the theoretical schools.

I wish to say further, in explanation, that the list of schools prepared by Dr. Billings for "Burdett's Hospital Annual" numbers only 49 in the United States, so that I feel quite sure my list included all the larger or more important schools, and a fair proportion of the small ones. Taking, then, 164 as the number upon which this report is based, I have 21 training schools with alumnæ associations or clubs, organised and in active operation, with constitution printed.

Ten training schools with alumnæ associations in process of organisation (constitution not printed); total, 31.

Seventy-eight training schools reporting no organisation, but showing interest; 55 not heard from; total, 133.

I have received copies of constitutions of 21 societies, and these I have divided into three classes: 1. Those organised and managed entirely by graduates which are alumnæ associations proper. 2. Nurses' clubs admitting to membership pupils of the school or graduates of other schools. 3. Religious societies, with a number of the officers, clergymen, or members of the training school board. Those included in the first class have practically a common object and the same form of government, differing, of course, in detail to meet the peculiar requirements of each society. The object of these associations is for the union of the graduates of the respective schools for mutual help and protection, to promote social intercourse and good fellowship, to provide friendly and pecuniary assistance in time of illness or death among members, and to advance the interest of the nursing profession.

Several societies pledge themselves to support the directory and school. Only those graduates in good standing in the profession are eligible for membership. Fees vary from five dollars to fifty cents. a year. The officers are a president, a vice-president, secretary, and treasurer, who are elected by ballot at the annual meeting to serve for one year, or until their successors are chosen.

Several of the societies have two vice-presidents, two secretaries (recording and corresponding), and two treasurers—the treasurer proper, not a member of the society, and a sub-treasurer, who is a member, and who performs the duties usually belonging to the treasurer. The duties of the treasurer proper are to have charge of the permanent or invested funds. The president presides at all meetings, and in her absence the duties of her office are performed by the vice-president. The duties of the secretaries I need hardly explain. In the majority of cases the officers form the executive committee, and transact all business of the association. They investigate all charges against any member, and she is given opportunity for defence before being expelled from the society. Several of the societies have a board of trustees, composed of the officers already mentioned, with several members who are elected at the same time. Still another way is to have a board of trustees composed of gentlemen, whose election is permanent, one member acting as treasurer already mentioned. This board invests the money of the association, and advises the officers of the society when necessary.

Where a society has received legacies or owns real estate, I should suppose a board of this kind would be necessary, but in small association, having only a contingent fund, it would seem to me better for the governing body to be composed entirely of members of the association, and the form of government to be as simple as possible. The executive committee, composed of the officers, would certainly be an easy and comprehensive plan to adopt when a society is forming.

Meetings are held monthly or quarterly, usually in the training school parlours. Notice of meetings and special business is sent by mail at least five days in advance by the secretary. Papers, discussions, lectures, and social intercourse are the usual features of the meetings. In a number of societies the election of officers is by ballot sent by mail; in others, voting is by members present, and the number necessary for a quorum differs.

The benefit fund is composed of all moneys not appropriated for the necessary expenses of the society obtained from initiation fees, yearly dues, donations and bequests.

One has both a sick fund and an annuity fund, the latter being made up of all that is left after expenses and benefits have been paid. One has a beneficial society that is a separate organisation, with an additional fee of six dollars, although all of the alumni are eligible for membership.

The amount allowed a sick member from the benefit fund also varies. In some cases the amount is limited to ten dollars a week: in another it is left to the discretion of the executive committee, and, when feasible, the nurse to be cared for at the hospital, the society bearing the expenses. Married members supported by their husbands are not entitled to benefits. Nearly all have honorary members, who pay no dues and have no vote, but are allowed to speak in meeting. One has honorary members who pay an annual fee of ten dollars and life members who pay fifty dollars.

There are minor points of interest in all the constitutions, but there are too many to enumerate at this time.

Of the clubs there are only four, and they differ from the alumnæ associations principally in their rules for membership. Pupils as well as graduates are eligible for membership, and can hold office, and the superintendent of the school is the president. Two of these clubs require no regular membership fee, but the expenses are met by voluntary contributions. These clubs have no benefit fund. One requires an annual fee of six dollars for graduates, and three for pupils; but this club has a benefit fund, its primary object being the care of sick members. There is one directory club, open to all graduates of regular schools, but with a membership of 89 names; only three are from outside schools. This, I will mention, is in connection with Rochester City Hospital. All of these clubs are wellorganised, but would, I think, be required to make some change in their constitution in order to be eligible for membership in a national alumnæ association.

Of the religious societies there are two, and, like the clubs, changes in their constitution would be necessary before membership in a national alumnæ could be considered. These societies are in connection with church hospitals, and should be classed properly with guilds. They unquestionably hold an important place in the schools with which they are connected.

There is a graduate nurses' club in the city, admitting to membership graduates from all schools in good standing. Its object is largely instructive and social, and it is exceedingly popular, and to nurses in the city fills the need, in a measure, of an alumnæ association in connection with their own schools.

I do not consider it necessary for me to even touch upon the advantages of alumnæ societies to nurses, my object being simply to show the material available for a national alumnæ; but in conclusion I want to urge upon the superintendents of schools that have not yet taken steps for organisation, the importance of immediate action in this matter. Organisation is the power of the age—without it nothing great is accomplished. All questions having the ultimate advancement of the profession are dependent upon united action for success The directory question; the uniform curriculum; the rejected probationer—every subject that concerns individual graduates, as well as schools, can only be reached through this channel.

The superintendent can do so easily what it is so very difficult for the graduates alone to accomplish, and she is the proper person to make the call for the first meeting. Even if she is not a graduate of the school, the hospital and the school are mutual points of interest to the older as well as the younger graduate, and all would recognise her as the proper leader in the movement. Do not wait for large numbers before taking action. A little society of ten members—let it be largely social if you will—forms a nucleus that time will develop. One superintendent, a New England girl, trained at the Massachusetts General, and who went West some years ago, "to grow up with the country," reports a small school, with an alumnæ of three members. That is the proper spirit.

If you have not a large school, make the most of your small one. Remember that it is only through organisation that individual members can be reached, and their co-operation in progressive movements be obtained, and that without their support and their good influences with the public we lose an immense power. I sincerely trust that when this Association of Superintendents holds its next annual meeting schools reporting "no organisation" may be very much in the minority.

Names of hospitals with which R. I. Hospital Providence Schools are connected in New York State: Bellevue, N. Y. City, Presbyterian, Mount Sinai, Charity, B. I., and Rochester City; Conn.: New Haven and Hartford; Penn.: University, Philadelphia-Blockley, Episcopal, Woman's, and Hahneman; Ohio: Cincinnati Tr. School (City); Cleveland: Homœopathic; Boston: City and Newton Cottage; Mass.: Worcesters City and Pittsfield (House of Mercy); Ind.: Johns Hopkins; D. C.: Children's and Garfield (Agitating); Chicago: Cook Co., Michael Reese, and St. Luke; Canada: Toronto General, and Kingston, Ontario.

### Wants and Workers.

[The attention of correspondents is directed to the fact that "Helps in Sickness and to Health" (Scientific Press, 428, Strand) will enable them promptly to find the most suitable accommodation for difficult or special cases.]

Would cut paper patterns for patchwork of various shapes be useful at any hospital or other institution? Address M. L. P., 5, Homefield Road, Wimbledon.

### The Book World for Women and Nurses.

[We invite Correspondence, Criticism, Enquiries, and Notes on Books likely to interest Women and Nurses. Address, Editor, The Hospital (Nurses' Book World), 428, Strand, W.C.]

Round the Red Lamp. A. Conan Doyle. (London: Methuen and Co.)

Forewarned is to be forearmed, and anyone finding objection to the subject matter of this book has the remedy in his own hands. The collection of stories entitled "Round the Red Lamp" are based in plot on the evidence of certain medical experiences. In challenging an objection to this, Mr. Conan Doyle speaks somewhat to the point. "A tale," he observes in his preface, "which may startle the reader out of his usual grooves of thought and shock him into seriousness, plays the part of the alterative and tonic in medicine, bitter to the taste, but bracing in its result. There are a few stories in this little collection which might have such an effect, and I have, so far, reserved them from serial publication. In book form the reader can see that they are medical stories, and can, if he, or she, be so minded, avoid them." But we can barely see that the writer has any occasion to apologise for the subject matter of these little tales. For they are delightful little stories, written in a fresh and healthy manner, and never offend the reader. It is true they are serious, reflective, and thoughtful, but never a bit *too* serious. "One cannot write of medical life and be merry over it," the author remarks tersely, and the tales, of which there are fifteen, are written principally concerning the graver side of human life. Out of the collection one may, perhaps, single "Sweethearts" as being the most attractive, but each and all are well worth the reading, and will certainly attain popularity with the public.

Hygiene. By J. Lane Notter, M.D., and R. H. Frith, F.R.C.S. (Published by Longmans, Green, and Co. Price 3s. 6d.)

This volume belongs to the series of Elementary Science Manuals published by Messrs. Longmans, and proposes to "present its facts and principles fully, briefly and yet in simple language suitable for both non-professional and professional readers." The non-professional readers will, we fear, fare badly, as some considerable knowledge of the various subjects treated of, as well as chemistry, meteorology, and vital statistics, will be required for the full appreciation of this book. On the other hand the professional reader will find it extremely useful for general reference, and especially for study immediately before an examination in sanitary science. In the chapter on food we are surprised to find repeated the old fallacy about beef-tea being "highly nutritive and restorative," instead of merely a stimulant. In other respects the information appears very complete and accurate, and the book can be cordially recommended to the earnest student.

### BOOKS RECEIVED.

Iliffe and Son.
"Nursing in a Nutshell." By a Doctor of Medicine."

Macmillan and Co.
"Theory and Practice of Counter Irritation." By H. Cameron Gilles, M.D.

William Clowes and Sons.
"The Volunteer Surgeon's Guide." By Surgeon-Captain Sleman.

Fannis and Co., Dublin.
"Ireland: Its Health Resorts and Watering Places."

**Periodicals and Pamphlets.**—Review of Reviews, Verulam Review, The Archæologist, The Therapist, Literary Digest, Revue Générale des Sciences, Invention, London, Brain—a Journal of Neurology, Gentleman's Magazine, Chapman's Magazine of Fiction, The Minster, Westminster Review, American Medico-Surgical Bulletin, Chicago Clinical Review, Humanitarian, Pears' Pictorial, English Illustrated Magazine, Sunday at Home, Leisure Hour, Boy's Own Paper, Girl's Own Paper, Penny Tales for the People, Indian Medico-Chirurgical Review, American Journal of Insanity, Charity Organisation Review. "Through the Long Light Watch." By M. L. B.

### French Schools for Trained Nurses: Their Origin and Organisation.

By Madame W. Vignal.

The primary instruction given at the Salpetrière Hospital School consists of reading, writing, and arithmetic. Dr. Bourneville, though not without considerable difficulty, has succeeded in combining the primary instruction with the first elements of the professional knowledge necessary for the male and female nurses. By this admirable arrangement they are prepared to subsequently follow the professional courses of lectures and the practical classes organised for their benefit. The teachers entrusted with the primary educational department choose the reading lessons from the five volumes of the "Nurse's Manual" (*Manuel de l'Infirmière*). The lectures given in the professional section are a verbal exposition of the information contained in this manual. In order to make the pupils expert in reading and writing, written copies of the *Manuel de l'Infirmière*, by Domville, are read, as well as the manual, "How to Treat Lunatics," published by the English Medico-Psychological Association.

Ever since the schools were founded in 1878, dictationshave been given on subjects related to the professional education necessary for the nurses, such as "The Conditions that must be fulfilled in order to obtain a Nurse's Diploma," "The signs of Death and the Steps to be taken for Burial" by Dr. Noir, "What is seen in a Hospital," "The Bath Service in Lunatic Asylums and Hospitals," "The Necessity of constantly supervising Epileptic Patients."

There is no special lecture-theatre or class-rooms; the classes are held in the evening in the clinical lecture-theatre of the Salpetrière Hospital, where also the professional lectures are given. The professors who kindly undertake to give these lectures are many of them well-known medical men, and others are former or present hospital house surgeons, but all are well educated and capable teachers.

In a former letter we have sketched the plan of instruction given to the Paris nursing staff, and will here only repeat that it is divided into courses of theoretical and practical lectures. The practical lectures are given in the Salpetrière General Infirmary by a *surveillante*, a *sous surveillante* and a *suppleante*: Cupping is also taught.

The Salpetrière school has given diplomas since 1882-1883. The following table will show how many have been granted each year during the last twelve years:

| | | | |
|---|---|---|---|
| 1882-1883 | ... 13 diplomas | 1888-1889 | ... 56 diplomas |
| 1883-1884 | ... 4 diplomas | 1889-1890 | ... 36 diplomas |
| 1884-1885 | ... 24 diplomas | 1890-1891 | ... 73 diplomas |
| 1885-1886 | .. 13 diplomas | 1891-1892 | ... 74 diplomas |
| 1886-1887 | ... 92 diplomas | 1892-1893 | ... 95 diplomas |
| 1887-1888 | ... 92 diplomas | 1893-1894 | ... 101 diplomas |

These statistics are evidence that the Salpetrière school is constantly progressing. The diplomas are gained by the Salpetrière nurses as well as by those of other hospitals who attend these classes.

(*To be continued.*)

### "The Hospital" Convalescent Fund.

### THE NURSES' BED.

The honorary secretaries acknowledge with thanks 2s. 6d. from Sister Moira, 2s. 6d. from Nurse Jarman, 10s. from Miss Mina Thompson, £1 from Miss Lucy Lees, and 2s. 6d. from Miss Dinwoodie (whom we thank for kind words of appreciation and promise of annual subscription), making £4 2s. 6d. received up to present date. The benefit tired nurses have gained from a rest and change at the seaside makes our task a grateful one. We arrange as far as possible to send nurses to localities as near their place of residence as possible, or to some Home chosen by themselves. In all cases our desire is to meet the requirements of each particular case, so that the rest and change may be both beneficial and enjoyable.

### For Reading to the Sick.

### PATIENCE.

**Motto.**

To wait and be patient soothes many a pang.

**Verses.**

How poor are they that have no patience!
What wound ever did heal except by degrees.
—*Shakespeare.*

O Heaven-born patience, source of peace and rest,
Descend; infuse thy spirit through my breast,
That I may calmly meet the hour of fate.
—*Mallet.*

God doth not bid thee wait
To disappoint at last;
A golden promise fair and great
In precept mould is cast.
Soon shall the morning gild
The dark horizon rim,
Thy heart's desire shall be fulfilled,
Wait patiently for Him.
—*F. R. Havergal.*

Oh! might we know! for sore we feel
The languor of delay,
When sickness lets our fainter zeal
Or foes block up our way.

Lord! Who Thy thousand years dost wait,
To work the thousand part
Of Thy vast plan, for us create
With zeal a patient heart.
—*Newman.*

God will make clear His purpose; I, at least,
Can wait in silence.
—*Plumtre.*

Let us be patient! These severe afflictions
Not from the ground arise,
But oftentimes celestial benedictions
Assume this dark disguise.
—*Longfellow.*

Endurance is the crowning quality,
And Patience all the passion of great hearts.
—*Lowell.*

**Reading.**

Patience is the guardian of faith, the preserver of peace, the teacher of humility, the cherisher of love. Patience governs the flesh, strengthens the spirit, sweetens the temper . . . . bridles the tongue, tramples upon temptations . . . . She comforts the poor and moderates the rich . . . . She teaches us to forgive those who have injured us, and to be the first in asking forgiveness of those whom we have injured. She delights the faithful and invites the unbelieving . . . . she is beautiful in either sex and every age. —*Bp. Horne.*

Let patience have her perfect work.—*S. James.*

A sick person does indeed need to have patience, and that of many kinds. Patience in bearing pain, patience in bearing all the privations of sickness, and all its many and accumulated trials. Patience to bear with those around him, their misunderstandings and obtuseness. Patience to bear with the circumstances of life, and his own peculiar lot. Patience to wait the appointed time until his change come.

It is indeed true, "ye have need of patience." The need seems to grow sorer as time goes on . . . Yet fear not if thus it is with you, "greater is He that is for you, than all they that are against you;" the battle is not yours but God's, and you shall be more than conquerors through Him that hath loved us. Tribulation worketh patience. A quiet, calm frame of mind, ever staying itself on God is the ground-work of patience; "in quietness and confidence shall be your strength." Stillness works patience; we must first get into the posture, and then the "God of patience" will be our refuge and strength.

"Sickness its trials and blessings."

Because thou hast kept the word of my patience, I also will keep thee from the hour of temptation.—*Rev. iii.* 10.

## Figures and Tables

**Figure f1:**